# Supplementary material for: Identification of photoperiod-induced specific miRNAs in the adrenal glands of Sunite sheep (Ovis aries)
Source: Front Vet Sci. 2022 Jul 22;9:888207. doi: 10.3389/fvets.2022.888207 (PMC9354845; doi:10.3389/fvets.2022.888207)
Supplement: Supplementary file 1 [file Data_Sheet_1.ZIP › Supplementary file/miRDeep2 file.zip!/miRDeep2 file/Know_Structure/SP42A3/oar-mir-539.pdf]

miRBase precursor : oar-mir-539
Total read count : 1
oar-miR-539-5p read count : 0
oar-miR-539-3p read count : 1
remaining reads : 0

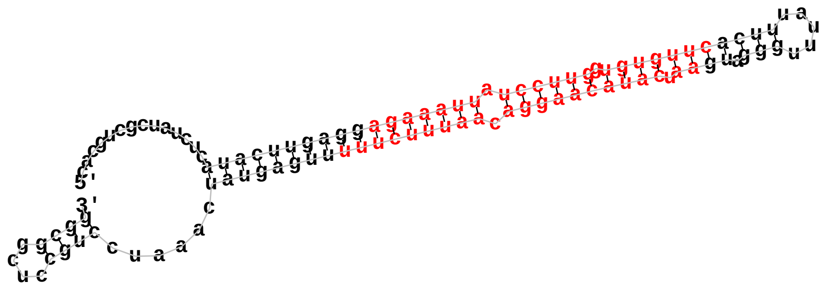

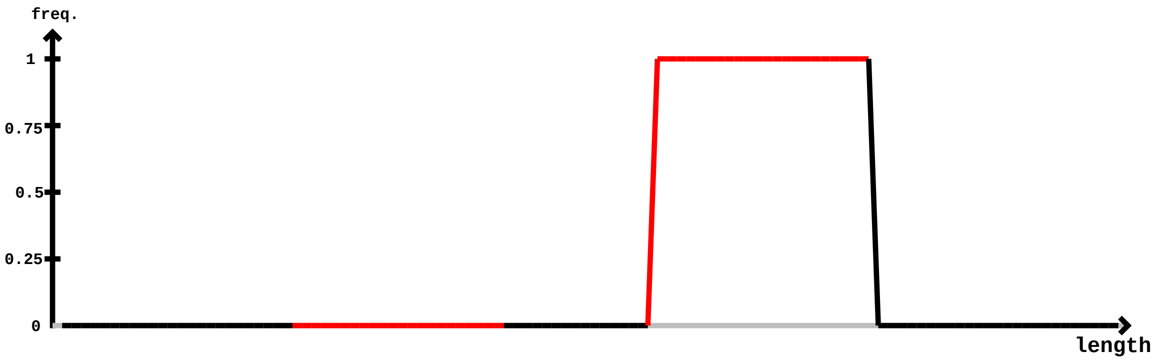


```
                                                                    oar-miR-539-3p
                                    oar-miR-539-5p
5'- cacgucgcuaucucauacuugaggagaaauuauccuugguguguucacuuuauuugggaugaaucauacaaggacaauuucuuuuugaguaucaaauccugccucggcgg   -3'   exp
    ..............(((((((((((((((((.((((((.(((((((((((.....))).)))).)))))))))).)))))))))))))))))......((((....))))    reads    mm    sample
    .............................................................aaucauacaaggacaauuucuuu..........................    1        0     seq
```
